# The National Pension Fund

**Published:** 1887-08-20

**Authors:** 


### THE NATIONAL PENSION FUND.

A correspondent asks how she may best help the Pension Fund. Personal propagandism is the best means; next to that the writing of letters to friends and the distribution at the same time to them of marked copies of The Hospital. If in addition to these methods, the friends written to could also be induced to write and send marked copies of The Hospital to their friends, and this process could be continually repeated in ever-widening circles, the National Pension Fund might be established almost immediately.

---

It will save a good deal of trouble if nurses and other hospital officials who propose joining the National Pension Fund will send their names and other particulars to *the Secretary of The Hospitals Association*, Norfolk House, Norfolk Street, W.C., and not to the Editor of The Hospital. Names continue to arrive in considerable numbers, but if those interested desire the Fund to be started before the end of the Jubilee year they must influence and interest all their friends immediately.

---

### From the Lady Superintendent of the St. Helena Home for Trained Nurses.

I send you the names of some of my nurses (17) who would like to join the Pension Fund, if when the scheme is promulgated it meets with their approval. By-and-bye, if not too late, I hope to send you more names. Owing to the nature of their work—private nursing—my nurses are widely scattered, and are often not at home for months together. Allow me to assure you of my own entire sympathy with the attempt to establish a National Pension Fund. It is a subject that has largely occupied my attention. No one can work long among nurses without becoming aware that few of them have any provision for the future to look forward to, while many have claims on their scanty earnings which make any attempt to lay by almost impossible, certainly quite inadequate to be of real use. I have always endeavoured to encourage thrift among my nurses, and to get them to invest what they could; but these individual efforts are very unavailing, and I have long come to the conclusion some public body must take the matter up, therefore I hail with pleasure the action of The Hospitals Association in trying to establish a National Pension Fund. From my own experience among nurses I could mention instances of piteous destitution and distress, where nurses have broken down from illness and had nothing to look forward to except private charity or the workhouse.

---

### From H. B.

Though I am neither a nurse or hospital official, I see The Hospital, and the idea of forming a National Pension Fund commends itself very much to my mind. I should feel obliged therefore if you would inform me, through its columns, how (in a small way) I could help the object forward. Nurses are certainly not too well paid, taking into account the arduous character of their many duties, and the risk and many unpleasantnesses incidental to them. Yet are their services (often by night as well as by day) always most kindly and cheerfully given. I am sure a Pension Fund would prove a great blessing to many in times of need, and an incentive to labour when in health and not needing assistance. Only let the desire to establish such a Fund be more generally made known, and I believe there are many among the public at large ready and willing to contribute towards such a much needed and worthy object.

---

### From the Lady Superintendent, Monsall Hospital, Manchester.

I enclose you a list of of my nurses. I have made two columns, as you will see, and have filled in under each name the amount the nurse in each case thinks she could subscribe. I am perfectly willing to subscribe to the Fund, and shall be glad if you will put my name on the list of those who most cordially approve of the movement. I wrote about two weeks ago asking for some definite information to give my nurses. I think the principal reason that so many hold back is that there is at present nothing definite—I mean as to what they are expected to do—for them to take hold of. Many of the people I have spoken to think highly of the proposal. I think it would be a good plan to print a large-size poster, with the objects of the Fund clearly stated, and rate of subscription.

---

### From a Nurse.

I am much interested in the discussion in The Hospital on the question, "A National Pension Fund for Trained Nurses." I should be thankful if such a Fund could be started. It has given me many anxious moments thinking what I should do, if by any cause I should be unable to work. I trust God will give me many years yet to work in the cause I love. The Home I joined four years ago sends notices to the effect in our papers, that no presents are to be accepted by any nurse, but if friends are pleased with the nurse's efforts, to send any donation to the Nurses' Pension Fund; but that Fund does not now exist. I am sure many patients and friends of our patients would often contribute if this Fund should be started. I would gladly become a subscriber, and I know a great many nurses would do so. Apologising for taking up your time.

---

### STRIKING INSTANCE OF HEREDITY.

*Lively Infant* (*aged* 4): "Papa, I saw such a funny beetle."

*Parent* (*enthusiastic amateur photographer*): "What did you do with him, my wee man?"

*Lively Infant:* "I photographed him in two positions, papa!"

